# A retrospective HEART risk score comparation of acute non-traumatic chest pain patients in an emergency department in Spain

**DOI:** 10.1038/s41598-021-02682-5

**Published:** 2021-12-01

**Authors:** Iris Nathalie San Román Arispe, Josep Ramón Marsal Mora, Oriol Yuguero Torres, Marta Ortega Bravo

**Affiliations:** 1 Centre d’Urgències d’ Atenció Primària (CUAP) Lleida, Institut Català de la Salut (ICS), Av. Prat de la Riba, 56, 25004 Lleida, Spain; 2 Research Support Unit Lleida, Fundació Institut Universitari per a la Recerca a l’Atenció Primària de Salut Jordi Gol i Gurina (IDIAPJGol), Lleida, Spain; 3 Unidad de Epidemiología del Servicio de Cardiología, CIBER of Epidemiology and Public Health (CIBERESP), Instituto de Salud Carlos III, Madrid, Spain; 4 Hospital Valld’Hebrón, Barcelona, Spain; 5 Research Group in Therapies in Primary Care (GRETAPS), Lleida, Spain; 6 Grupo Transversal de Urgencias y Emergencias, IRB Lleida, Lleida, Spain; 7 Servicio de Urgencias del Hospital Universitario Arnau de Vilanova, Lleida, Spain

**Keywords:** Cardiology, Diseases

## Abstract

Non traumatic chest pain is the second most common cause of attention at the Emergency Departments (ED). The objective is to compare the effectiveness of HEART risk score and the risk of having a Major Adverse Cardiovascular Event (MACE) during the following 6 weeks in ‘Acute Non-traumatic Chest Pain’ (ANTCP) patients of an ED in Lleida (Spain). The ANTCP patient cohort was defined using medical data from January 2015 to January 2016. A retrospective study was performed among 300 ANTCP patients. Diagnostic accuracy to predict MACE, HEART risk score effectiveness and patient risk stratification were analysed on the ANTCP Cohort. HEART risk score was conducted on ANTCP Cohort data and patients were stratified as low-risk (n = 116, 38.7%), moderate-risk (n = 164, 54.7%) and high-risk (n = 20, 6.7%); differently from the assessment performed by 'Current Emergency Department Guidelines’ (CEDG) on the same patients: low risk and discharge (n = 56, 18.7%), medium risk and need of complementary tests (n = 137, 45.7%) and high risk and hospital admission (n = 107, 35.7%).The incidence of MACE was 2.5%, 20.7% and 100% in low, moderate and high-risk, respectively. Discrimination and accuracy indexes were moderate (AUC = 0.73, 95% confidence interval: 0.67–0.80). Clustering moderate-high risk groups by MACE incidence showed an 89.5% of sensitivity. Data obtained from this study suggests that HEART risk score stratified better ‘acute non-traumatic chest pain’ (ANTCP) patients in an Emergency Department (ED) compared with ‘Current Emergency Department Guidelines’ (CEDG) at the Hospital Universitari Arnau de Vilanova (HUAV). HEART score would reduce the number of subsequent consultations, unnecessary admissions and complementary tests.

Trial registration: Retrospectively registered.

## Introduction

‘Acute Non-Traumatic Chest Pain’ (ANTCP) is the second cause of consultation in Emergency Department (ED) services in industrialized countries. The 70% of Acute Myocardial Infarctions (AMI) present as non-ST elevation acute coronary syndrome (NSTEACS). The appropriate management of the ANTCP is of great importance to avoid unnecessary costs and inappropriate hospital admissions^1–3^.

The diagnosis of a Major Adverse Cardiac Event (MACE) in the context of NSTEMI (Non ST Elevation Myocardial Infarction) is complicated, both due to its own complexity and clinical ambiguity in addition to the fact that European Society of Cardiology’s (ESC) recommendations for NSTEMI diagnosis are imprecise^4^. For this reason, it is crucial to establish a guide for the correct diagnosis and prognosis of MACE after the first consultation for ANTCP in the ED.

Of all the potential NSTEMI, only 20% resulted in coronary syndrome. Approximately half of the patients admitted for suspected Acute Coronary Syndrome (ACS) and subjected to diagnostic cardiac tests were not finally diagnosed with ACS. Tests performed on patients not diagnosed with ACS cost up to 10 billion dollars annually, meaning an estimated cost of 3000–6000 dollars per patient. Chest pain was produced due to cardiac cause in only 10% of those patients, according to studies carried out in the United States^5^.

In addition, between 5 and 10% of patients who were discharged from the ED because it was considered that the ANTCP origin was not coronary, presented an Acute Myocardial Infarction^5^.

In Spain, according to a study published in 2013^6^, ACS is one of the main causes of morbidity, mortality and expense. There are approximately 120,000 cases of ACS per year, 50,000 of whom are hospitalized (41.66% patients/year) with NSTEMI^4^.

TIMI^7^ and GRACE^8,9^ risk scales are those recommended by the latest guidelines of the European Society of Cardiology^6^ and the National Institute for Health and Care Excellence (NICE)^10^, especially for patients with suspected NSTEMI. These scales were developed from studies of patients already diagnosed with ACS, and are useful for those patients who have a high probability of presenting an ACS^9,11^.

The HEART scale provides an excellent determination of the MACE risk in the ANTCP consultations for 6 weeks^10,12–15^. The HEART score classified patients as low risk with a sensitivity of 99.5% and a negative predictive value of 99.6%,identified it as high risk with a specificity of 90.9% for 30-day MACE, according to recent multinational validation studies^9,15–18^. In November 2020 a prospective study of HEART observing that 97.5% of low-risk patients had no death from AMI within 1 year, with a 7.0% reduction in hospitalization/year^19^.

HEART scale is currently validated for use in emergency medical services over TIMI or GRACE according to some of the latest recommendations^9,15,18^. Similar studiesperformed in Spain were not found, therefore this studywas carried out with the objective of compare the usefulness of HEART scale for diagnose, stratify the risk of patients and predict MACE in the next 6 weeks after the consultation in the Emergency Service of our hospital in this population.

## Methods

### Design

Retrospective observational cohort study, including patients who attended the HUAV Emergency Department with ANTCP, between January 2015 and January 2016.

The sources of information were the computerized registry of the clinical file (SAP) of the Hospital Universitary Arnau de Vilanova and the Institut Català de la Salut Primary Care medical registry (e-CAP) of Lleida-Spain.

### Sample size

An inclusion of 300 patients in the sample is the minimum to determine a difference in the C statistic for the ROC curves of 0.08, assuming a C statistic for the HEART scale of 0.83 with a type I error of 0.05. A representative sample of 300 patients was obtained from the study population through consecutive sampling, including those patients who met all the inclusion criteria and none of the exclusion criteria.

### Inclusion criteria

We selected patients older than 18 years old who had non-traumatic acute chest pain lasting more than 5 min, with clinical characteristics compatible with suspicion of ACS according to the guidelines of the European Society of Cardiology (ESC), without evidence of a heart attack.

Of these patients, we included in the sample those ones who had all the complete data in the clinical record of the HUAV electronic registry for the performance of the HEART scale and a 6-week retrospective follow-up after the consultation.

### Exclusion criteria

Patients for whom insufficient information was available to carry out the minimum six-week follow-up based on administrative data; patients who lacked the necessary data to apply the HEART clinical risk score, patients that modified during the 6-week follow-up any of the risk factors for an acute cardiovascular event(e.g., initiation of cocaine use or any other cardioactive or vasoactive drug that produces and/or favors the development of a MACE in a direct way) and those patients who did not stay in Lleida-Spain, as it could alter the follow-up.

### Variables

The HEART risk score is an acronym for its components: History (the characteristics of chest pain compatible with acute coronary syndrome), ECG, Age, Risk factors, and Troponin.

Each one of these factors was scored with 0, 1 or 2 points. According to these items, the patients were stratified into three risk levels: low risk (≤ 3 points), moderate risk (4–6 points) and high risk (≥ 7 points). The diagnostic accuracy of the HEART score (0–10) was calculated to predict the primary outcome of major adverse cardiac events (MACE) for 6 weeks after a consultation at the emergency room.

The TnI used in the HUAV was scored as follows: 0 points = normal limit < 0.01 ng/mL, 1 point = 1–3 times the normal limit 0.01 ng/mL-0.03 ng/mL, 2 points = more than 3 times normal limit ≥ 0.04 ng/mL (We used the ultra-sensitive Troponin T, not corrected for haemolysis. The upper limit of the reference range corresponds to the 99th percentile of the population studied by our method^19,20^.

Also, other independent variables were taken into account in addition to the variables of the HEART scale: gender, diagnosis at discharge from the emergency room or at hospital admission, performed cardiac tests, coagulation and other blood test used in the diagnostic process. It was recorded the number of patients discharged, the number of patients that remained in hospital for observation and the number of patients that were admitted as well as the diagnostic resources employed.

The dependent variable was MACE, which includes: Deaths from all cardiac causes, Acute Myocardial Infarction (AMI), Coronary Artery Bypass Grafting (CABG), Significant Stenosis (> 50%) with Conservative Treatment (SSCT), Percutaneous Coronary Interventionism (PCI), both at the time of emergency as well as during the 6 weeks following the consultation.

### Regular practice and follow-up

The sample of 300 patients, who received regular medical care at the HUAV ED, was registered in the SAP and in the e-CAP file archives. “Standard medical practice” in the ED was the chosen one to be performed by the physicians who took care of those patients who presented ANTCP; therefore the treating physicians assessed their risk without using the HEART score.

Based on these data and according to the care route followed, patients were divided into 3 groups: The first group comprehended those patients discharged due to the diagnosis of MACE at the time of consultation; in the second group—of patients who remained under observation in the ED, MACE was undoubtedly diagnosed at the time of consultation and further complementary tests were required; and the third group was composed by those patients admitted into the Hospital due to a diagnosis and/or high suspicion and/or high risk of MACE.

From the sample of 300 patients to whom the HEART scale was applied based on the data from the SAP and the e-CAP computer registry, depending on the HEART score, three risk groups arise: Low, Medium and High Risk.

The group of patients who consulted for ANTCP and who were discharged with a diagnosis other than MACE was followed up for 6 weeks (time of the predictive value of the HEART risk score) by reviewing the eCAP and SAP files.

Complementary tests, interventions or cardiac study resources that were objectively used, such as angiography, 2 or more Troponins, ergometry and/or others, were quantified from the group of patients admitted or under observation in the hospital.

Diagnoses were recorded at discharge from the first DTANT consultation, after hospital stay or admission.

### Ethical aspects

This study was approved on 31st March 2016, minute 3/2016 by The Ethical Committee (CEIm) of Hospital Universitari Arnau de Vilanova de Lleida de la Gerència Territorial ICS Lleida, Alt Pirineu i Aran—GSS (approval code: CEIC‐1605).

### Statistical analysis

Continuous variables are expressed as mean and standard deviation (SD) and categorical variables as absolute and relative frequency. Variables were compared using Chi-square test for qualitative variables and the non-parametric Kruskal–Wallis test for continuous variables. To determine the effect of HEART score on MACE, a logistic regression was adjusted by age and gender. The area under the ROC (Receiver Operating Characteristic) curve was used as a discrimination model and accuracy index. Hosmer–Lemeshow C-test was applied to assess model’s calibration by assembling patients by similar model outputs. The sensitivity, specificity, Positive Predictive Value (PPV) and Negative Predictive Value (NPV) were studied concurrently. A significance level of 0.05was considered (α = 0.05).

SPSS21 was used for data management and statistical analysis.

### Ethics approval and consent to participate

Conforming to Law 14/2007, July 3, the Biomedical Research Regulations in Spain, Article 3-m, this study corresponds to an «Observational Study»: study carried out on individuals in respect of whom the treatment or intervention to which they may be subjected is not modified nor is any other guideline prescribed that could affect their personal integrity to be subject to an observational study. The patients received the optimal treatment according to the current guidelines of the Hospital Universitari Arnau de Vilanova of Lleida—Spain. This study was approved on 31st March 2016, minute 3/2016 by The Ethical Committee (CEIm) of Hospital Universitari Arnau de Vilanova de Lleida de la Gerència Territorial ICS Lleida, Alt Pirineu i Aran—GSS (approval code: CEIC‐1605), and informed consent was not requested from the patients in consonance with Organic Law 15/1999 (December 13) on the Protection of Personal Data (LOPD) in force in Spain on that date *. In addition, due to the nature of this study, the need for informed consent was waived by The Ethical Committee (CEIm) of Hospital Universitari Arnau de Vilanova de Lleida de la Gerència Territorial ICS Lleida, Alt Pirineu i Aran—GSS (approval code: CEIC‐1605). Specific information was collected to answer the research question. * This law was replaced by the Data Protection Regulation (RGPD) and European regulation, relative to the protection of natural persons with respect to the processing of personal data and the free circulation of data, which entered into force on May 24, 2016 and is applicable as of May 25, 2018.

## Results

The group of ‘Acute Non-Traumatic Chest Pain’ (ANTCP) cases was selected from the database. The total number of patients who attended the Emergency Department (ED) of the Hospital Universitari Arnau de Vilanova (HUAV) was of 2.127 patients. To more precisely define the ANTCP patient cohort, exclusion/inclusion criteria were applied into the ANTCP cases group; and the ANTCP patient cohort was established with a total of 535 patients, from whom 300 patients were selected using a consecutivesystematic sampling.

**Figure 1:**
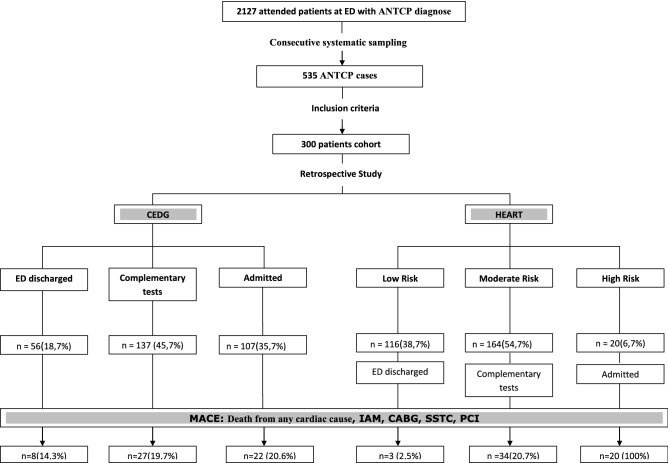
Patient flow chart for patients included in current comparison of performance of the HEART score and ‘Current Emergency Department Guidelines’ (CEDG).

ANTCP patient cohort was first assessed according to ‘Current Emergency Department Guidelines’ (CEDG).This routine classified the patients into three groups: Those discharged from the ED (n = 56, 18.7%), those who required complementary tests (n = 137, 45.7%) and those being admitted in the Hospital (n = 107, 35.7%). HEART risk score was conducted on ANTCP patient Cohort data and patients were stratified as low-risk (n = 116, 38.7%), moderate-risk (n = 164, 54.7%) and high-risk (n = 20, 6.7%). Differences were observed on ED discharged and low-risk groups, as well as in admitted and high-risk groups. This could be related with the diagnosis routine and it clearly showed that HEART risk score would be a great tool for early classification of the ANTCP patients at ED (Fig. 1).

At the Hospital ED 107 patients (35.7%) were admitted due to high risk of MACE. Of those, just 22 (20.6%) developed a MACE, in contrast with the HEART score, were 20 patients (6.7%) were classified as high risk patients and the 100% developed a MACE (Fig. 1).

The average age of ANTCP patient Cohort was58.3 ± 12.5 years old and there were 127 female patients (42.3%) and 173 male patients (57.7%). Most of them were in the ‘45–64’age group (n = 141, 47%).

Additionally, the results of components of HEART’s risk score that were evaluated are (*p* < 0.001, chi-square test): Most of the patients (60%) had moderate clinical suspicion or clinical history of chest pain suggestive of MACE, predominantly (71.3%) normal ECG without nonspecific alteration in repolarization; 55% of patients presented one or two risk factors, the most frequent being hypercholesterolemia (n = 155, 51.7%), Diabetes Mellitus (n = 118, 39.3%) and smoking (n = 118, 39.3%); the vast majority of troponin levels (89.7%) were within the normal range; and only 8 patients had troponin levels three or more times normal (2.7%) (Table 1).

**Table 1:** Baseline patient characteristics by HEART risk score.

Variables	Total (N = 300)		Low-risk (N = 119)		Moderate-risk (N = 164)		High-risk (N = 17)		*p*^a^
	N	(%)	N	(%)	N	(%)	N	(%)	
**Gender**									0.116
Female	127	42.3	56	4 7.1	66	40.2	5	29.4	
**Age**									< 0.001
< 45	54	18	40	33.6	14	8.5	0	0	
45–64	141	47	62	52.1	77	47	2	11.8	
≥ 65	105	35	17	14.3	73	44.5	15	88.2	
**History**									< 0.001
Slightly suspicious	20	6.7	20	16.8	0	0	0	0	
Moderately suspicious	180	60	91	76.5	89	54.3	0	0	
Highly suspicious	100	33.3	8	6.7	75	45.7	17	100	
**ECG**									< 0.001
Normal	214	71.3	105	88.2	109	66.5	0	0	
Non-specific repolarization disturbance	72	24	14	11.8	53	32.3	5	29.4	
Significant ST depression	14	4.7	0	0	2	1.2	12	70.6	
**Risk factors**									
Hypercholesterolemia	155	51.7	18	15.1	120	73.2	17	100	< 0.001
HTN	86	28.7	19	16	56	34.1	11	64.7	< 0.001
DM	118	39.3	22	18.5	90	54.9	6	35.3	< 0.001
Smoking	118	39.3	28	23.5	84	51.2	6	35.3	< 0.001
Positive family history	35	11.7	8	6.7	21	12.8	6	35.3	< 0.002
Obesity (BMI ≥ 30)	64	21.3	20	16.8	39	23.8	5	29.4	< 0.102
**Troponin**									< 0.001
≤ Normal limit	269	89.7	119	100	143	87.2	7	41.2	
1 − 3 × normal limit	23	7.7	0	0	19	11.6	4	23.5	
≥ 3 × normal limit	8	2.7	0	0	2	1.2	6	35.3	

*ECG* Electrocardiogram, *HTN* Hypertension, *DM* Diabetes mellitus, *BMI* Body mass index.

^a^Chi-square test.

We applied the HEART scale to the cohort of patients who consulted for ANTCP and were suspected of NSTEMI, thus they remained in the Hospital ED while further complementary tests were performed and/or were assessed by other specialists, and we obtained the following data: Low-risk patients stayed in the ED for a mean of 7.24 h, and high-risk patients for a mean of 12.5 h (Table 2).

**Table 2:** The mean of stay hours in ED.

	Total (N = 300)		Low-risk (N = 116)		Moderate-risk (N = 164)		High-risk (N = 20)		*p*
	N	(%)	N	(%)	N	(%)	N	(%)	
The mean of stay hours in the ED room	300	9.6	116	7.24	164	11.02	20	12.5	< 0.001^a^
**Diagnostic ICD 10**									
Precordial pain	33	11	0	0	24	14.6	9	52.9	
Atypical chest pain	55	18.3	19	16	34	20.7	2	11.8	
Musculoskeletal chest pain	70	23.3	54	45.4	16	9.8	0	0	
Nonspecific chest pain	73	24.3	28	23.5	43	26.2	2	11.8	
Angina	10	3.3	2	1.7	8	4.9	0	0	
Others^b^	59	19.7	16	13.4	39	23.8	4	23.5	
Return patient^c^	0.62	0.9	0.19	0.5	0.88	0.9	1.06	1.2	< 0.001^d^
MACE	57	19	3	2.5	34	20.7	20	100	< 0.001^a^
Incidence of MACE	57	0.19	3	0.025	34	0.20	20	1	

Final diagnosis. Reconsultation for ANTCP. MACE estimation within 6-weeks.

*p p*-value, *MACE* Major adverse cardiovascular events.

^a^Chi-square test.

^b^Other diagnosis: gastric pathology (32%), hepatobiliary pathology (19%), pulmonary pathology (13%), pancreatic pathology (11%), pleura pathology (7%), pericardial disease (5%), others (13%).

^c^Number of return patient consultations after discharge within 6-weeks.

^d^Non-parametric Kruskal–Wallis test.

The diagnostic coding according to ICD 10 assigned by ED physicians who attended patients who were visited by ANTCP was mostly “Nonspecific chest pain” (26.2%), closely followed by “Musculoskeletal chest pain” (23.3%). “Angina-type chest pain” (3.3%) was the least common. Within the diagnosis of “others”, gastric pathology (32%) predominated, followed by hepatobiliary pathology (19%). Most of the patients classified as Low Risk according to HEART were discharged (45.4%) with the diagnosis of "Musculoskeletal chest pain"; On the other hand, of those classified as High Risk, more than a half (52.9%) were discharged with the diagnosis of “Precordial pain” (Table 2).

The number of patients who returned for to consultate for ANTCP within 6 weeks was higher in those classified as High Risk (1.2%) according to the HEART scoring scale. A directly proportional relationship between the number of patients who returned for to consultate for ANTCP within 6 weeks and risk group according to the HEART scale was targeted: low risk = 0.19; moderate risk = 0.88; high risk = 1.06 (*p* < 0.001, Kruskal–Wallis test) (Table 2).

The incidence of MACE applying the HEART scale was 2.5%, 20% and 100% at low, moderate and high risk, respectively (Table 2). Discrimination and precision indices were moderate (AUC = 0.80, 95% confidence interval: 0.74–0.87, *p* < 0.001, Chi-square test). The most frequent MACE (*p* < 0.001, Chi-square test) in the high-risk group were myocardial infarction (AMI; 42.2%) and significant stenosis with conservative treatment (ESTC; 34.2%) unlike the Low-Risk group, in which the most frequent MACE was acute coronary syndrome that required Percutaneous Coronary Interventionism (PCI; 2.7%) and AMI (0.8%) (Table 3).

**Table 3:** MACE according to the stratification made by HEART scale.

	Low risk (n = 116)		Moderate risk (n = 164)		High risk (n = 20)		Total (n = 300)		*p*^a^
	N	n (%)	N	n (%)	N	n (%)	N	n (%)	
Death from any cardiac cause	116	0 (0%)	164	2 (1.2%)	20	0 (0%)	300	2 (0.7%)	0.407
IAM	116	1 (0,8%)	164	13 (7.9%)	20	10 (42.2%)	300	21 (7%)	< 0.001
CABG	116	0 (0%)	164	4 (2.4%)	20	2 (11.8%)	300	8 (2.7%)	0.094
SSTC	116	0 (0%)	164	8 (4.9%)	20	6 (34.2%)	300	14 (4.7%)	< 0.001
ICP	116	2 (1,7%)	164	7 (4.3%)	20	2 (11.8%)	300	12 (4%)	0.119
MACE	116	3 (2,5%)	164	34 (20.7%)	20	20 (100%)	300	57 (19%)	< 0.001

*MACE* Major adverse cardiovascular events, *MI* Myocardial infarction, *CR* Coronary revascularization, *SSCT* Significant stenosis with conservative treatment, *p p*-value.

^a^Chi-square test.

According to the stratification made by HEART scale, the risk of MACE in moderate/high-risk patients was 5 times higher than in low-risk patients; this was adjusted by age and sex with an OR = 5.28 (95% CI: 2.1–13.4) and *p* < 0.001(Table 4).

**Table 4:** Risk of MACE adjusted by age and sex.

	OR	CI 95%	*p* value
High risk (> 3points)	5.28	(2.1–13.4)	< 0.001
Age	1.03	(1–1.1)	0.028
Sex (female)	1.03	(0.6–1.9)	0.921
Hosmer & Lemeshow (calibration)	11.8		0.160
ROC curves (discrimination)	0.73	(0.67–0.8)	< 0.001

The AUC of HEART score was 0.73 (95% confidence interval 0.67–0.8). High Risk ORwas 5.28 (95% confidence interval) and a *p*-value < 0.001, the age was the main risk factor with a OR 1.03 *p*-value 0.028.

*MACE* Major adverse cardiovascular events, *OR* Odds ratio, *CI* Confidence interval.

A univariate logistic regression was used to model the relationship between HEART and MACE scores. Sensitivity was of 95% (95% CI: 87.5–99.0and specificity of47% (95% CI: 40.2–52.8) both with a 95% CI. Positive predictive value was of 28.9% and negative predictive value was 97%, (Table 5).

**Table 5:** Performance characteristics of HEART risk stratification strategy.

Risk stratification strategy	6-weeks MACE		Total (n)
	No (n)	Yes (n)	
Low-risk (≤ 3)	113	3	116
Moderate/high-risk (> 3)	130	54	184
Total	243	57	300
Sensitivity (%)	95.0	51/57	95%CI: 87.5–99.0
Specificity (%)	47.0	113/243	95%CI: 40.2–52.8
Positive predictive value (%)	28.9	51/181	
Negative predictive value (%)	97.0	113/119	

*MACE* Major adverse cardiovascular events.

## Discussion

HEART risk score is a useful, easily to apply tool in the diagnosis and prognosis of MACE for ED professionals. Moreover, HEART scale showed greater usefulness in risk classification and diagnosis of MACE in the cohort of patients with ANTCP and an initial ECG without ST elevation, compared to CEDG.

According to the usual care in ED of patients with ANTCP suspected of NSTEMI a first group was discharged due to the diagnosis of MACE at the time of consultation n = 56 (18.7%) and had MACE n = 8 (14.3%), in contrast to HEART scale that made a better risk stratification in which n = 116 (38.7%) were classified as low risk and had MACE n = 3 (2.5%).

Of the group of patients considered high risk according to the CEDG of the ED, were admitted into the Hospital n = 107 (35.7%) due to diagnosis and/or high risk of MACE, of which ones had MACE n = 22 (20.6%), unlike the HEART scale, which showed better performance in classifying high-risk patients for MACE n = 20 (6.7%) and had MACE n = 20 (100%).

It is convenient to highlight a better diagnostic power in patients who were discharged from ED and had MACE in the next 6 weeks after consultation. According to CEDG in ED, 18.7% of patients were discharged (n = 56), 8 of whom (14.3%) had MACE within the following 6 weeks. Otherwise, using HEART risk score 116 patients were stratified as low risk and discharged, 3 (2.5%) of which had MACE, suggesting an important difference. Therefore, the classification of patients with HEART risk score and prediction of the primary outcome of MACE were more accurate compared to CEDG. These results had already been described previously by different studies^11,16,18^.

Of the group classified as low risk according to HEART and who’s had MACE, none died of cardiac causes. It is evident that the incidence and risk of MACE after 6 weeks in low-risk patients stratified according to the HEART scale is significantly minor and safer than with the usual care following the guidelines of the ESC in ED (according to most of the studies^9,15–19,21^). As few studies state^9,11^ probably the prognostic risk of HEART would improve even more if the scale had been applied prospectively and/or if we had shortened the number of days to 30 days instead of 6 weeks, but that would be a matter for other clinical trials.

The high diagnostic performance of HEART scale is reinforced by results regarding the incidence of MACE according to risk groups, which are 0.025%, 0.20% and 1% in low, moderate and high risk, respectively (Table 2). Also, the diagnostic power of HEART scale in terms of the risk of MACE in moderate/high-risk patients stands out which was 6 times higher than in low-risk patients; This was adjusted by age and sex (OR = 5.28, 95% confidence interval: 2.1–13.4, *p* < 0.001, Table 4); with discrimination indices and precision being moderate (AUC = 0, 87, 95% confidence interval: 0.80–0.90, *p* < 0.001).

HEART scale takes into account clinical history of chest pain, as well as other clinical characteristics that are easy to handle and available to most EDs in Spain. It was observed in this study that the different components that make up HEART cardiovascular risk assessment are well constructed since each one of them has statistical significance (*p* values less than 0.01, Table 1).

The mean hours of stay in ED are higher than that required if patients had been stratified with HEART scale. Those patients at low risk with an average of 7.24 h and in those at high risk with an average of 12.5 h would have benefited from a home discharge or a Hospital admission without compromising patients’ health (Table 2).

An adequate stratification of moderate-high-risk patients could avoid follow-up consultations while favoring an optimal use of healthcare resources.

Patients classified according to HEART high-risk scale, consulted on average 1.06 times more, in contrast to the 0.88 times of the moderate risk ones and the 0.19 visits of patients with a low risk. Besides, late treatments are more expensive and, at the same time, have a higher risk of morbidity and mortality for patients at risk with a score > 3 who were not treated in due time.

Due to the high NPV (97%) of HEART risk scale added to its high diagnostic sensitivity (95%), its systematic use in EDs could improve the efficacy in the care of patients who come to ED with ANTCP and suspected NSTEMI on first ECG.

Although a representative sample has been obtained that allows the results to be extrapolated, this study was carried out only in a Hospital with its own healthcare and population characteristics, thus obtained findings must be interpreted with care. To reinforce these data, it will be necessary to carry out a study that covers more health care centers.

Another weakness of the study is the loss of patients during selection, since those patients who did not meet inclusion criteria were excluded from the study, as well as those who could not be followed up during the following 6 weeks due to lack of data or impossibility of contact.

In order to minimize this possible bias, a comparative analysis was made between the patients who were followed up and those who were not. When we detected clinically relevant differences, weights were assigned based on these differences. In general, the proportion of patients excluded from the final analysis was compared with the sample of patients that we did include in the analysis. This comparison was made at the level of the baseline variables and in case of determining important differences, the data of the included patients were weighted based on weights. Eights were developed using inverse probability weighting algorithms that were validated and applied in different observational studies.

## Conclusions

These results suggest that HEART risk score better stratified ‘Acute Non-Traumatic Chest Pain’ ANTCP patients in Emergency Department (ED) compared with ‘Current Emergency Department Guidelines’ (CEDG) at the Hospital Universitari Arnau de Vilanova (HUAV). This retrospective study showed a very high effectiveness to stratify ANTCP patients in low/moderate/high-risk groups and determined the risk of MACE during the following 6-weeks.

Consequently, the potential implementation of HEART score in EDs would allow better care for patients who attend an ED with ANTCP and are suspected of NSTEMI through adequate risk stratification of MACE. This would provide a safer discharge to those with low risk without compromising patients’ health. Additionally, reduces the use of unnecessary additional health resources, the length of hospital stays, avoids unjustified admissions and reduces the number of follow up consultations. Further, it could imply better care for moderate-high risk patients, who would benefit from timely diagnosis and treatment, reducing morbidity and mortality. Plus, health resources usage would be lowered due to the cut in follow up consultations and/or assistance for complications product of early diagnosis and pertinent treatment.

This research contributes to highlight the advantages of the HEART risk score by suggesting a greater performance over other options as a reliable, quick and easy-to-use diagnostic stratification method in the assessment of patients with non-traumatic acute chest pain. We suggest that its systematic use would allow less use of health resources.

## Data Availability

The datasets generated and/or analyzed during the current study are not publicly available, the data was saved together with the clinical data of the study to be able to carry out the follow-up, but they are available from the corresponding author upon reasonable request.

## Abbreviations

ANTCP: Acute non-traumatic chest pain
MACE: Major adverse cardiac event
ESC: European Society of Cardiology
ECG: Electrocardiogram
NSTEACS: Acute coronary syndrome without ST segment elevation
NSTEMI: Non ST elevation myocardial infarction
ED: Emergency department
HUAV: Hospital Universitari Arnau de Vilanova
HEART: History, ECG, age (in English Age), risk factors (in English Risk factor’s) and troponin
CEDG: Current emergency department guidelines
AMI: Acute myocardial infarction
CABG: Coronary artery bypass grafting
SSCT: Significant stenosis with conservative treatment
PCI: Percutaneous coronary interventionism
GRACE score: Global registry of acute coronary events
TIMI score: Thrombolysis in myocardial infarction
ACS: Acute coronary syndrome
NICE: National Institute for Health and Care Excellence
